# Using the Facebook Advertisement Platform to Recruit Chinese, Korean, and Latinx Cancer Survivors for Psychosocial Research: Web-Based Survey Study

**DOI:** 10.2196/11571

**Published:** 2019-01-10

**Authors:** William Tsai, Daisy Zavala, Sol Gomez

**Affiliations:** 1 Department of Applied Psychology New York University New York, NY United States; 2 Department of Psychology California State University San Marcos San Marcos, CA United States

**Keywords:** ethnic minority cancer survivor, Facebook recruitment, Korean cancer survivor, Chinese cancer survivor, Latinx cancer survivor, mobile phone

## Abstract

**Background:**

Ethnic minority cancer survivors remain an understudied and underrepresented population in cancer research, in part, due to the challenge of low participant recruitment rates. Therefore, identifying effective recruitment strategies is imperative for reducing cancer health disparities among this population. With the widespread use of social media, health researchers have turned to Facebook as a potential source of recruitment.

**Objective:**

We aimed to evaluate the feasibility and effectiveness of purchasing ads on Facebook to recruit Chinese, Korean, and Latinx cancer survivors residing in the United States. We assessed their experience with participating in a Web-based survey and their interest for future research.

**Methods:**

We showed 5 purchased ads in English, simplified Chinese, traditional Chinese, Korean, and Spanish on Facebook. Participants who clicked on the Facebook ad were directed to the study website and asked to submit their emails to receive the link to the 30-minute Web-based survey. Inclusion criteria included being of Asian or Latinx heritage, age ≥18 years, having a cancer diagnosis, and being within 5 years of cancer treatment. Participants who completed the survey were sent a US $10 Walmart eGiftcard.

**Results:**

The Facebook ads were shown for 48 consecutive days for a total spending of US $1200.46 (US $25/day budget). Overall, 11 East Asian and 15 Latinx cancer survivors completed the study, resulting in an average cost per participant of US $46.17. The East Asian and Latinx cancer survivors did not significantly differ in age, years lived in the United States, education level, generation status, and time since diagnosis. However, Latinx cancer survivors were marginally more likely to have limited English proficiency and lower annual income than East Asian cancer survivors. Both Latinx and East Asian cancer survivors reported that they enjoyed participating in this study and indicated an interest in participating in future psychosocial research studies.

**Conclusions:**

The use of Facebook ads successfully resulted in the recruitment of East Asian and Latinx cancer survivors with different cancer diagnoses who reside in various geographic regions of the United States. We found that East Asian and Latinx cancer survivors recruited through Facebook were interested in participating in future psychosocial research, thereby providing support for the feasibility and effectiveness of using Facebook as a source of recruitment for ethnic minority cancer survivors.

## Introduction

Because ethnic minority cancer survivors experience cancer health disparities along the entire cancer control continuum [1], initiatives such as the Healthy People 2020 [2] have called for greater attention and efforts to increase the representation of ethnic minorities in cancer research. However, they remain an understudied and underrepresented population in cancer research in part owing to the persistent challenge of low recruitment rates. Despite researchers’ persistent efforts, traditional recruitment strategies such as utilizing physician referrals, purchasing newspaper ads, and using cancer registries have resulted in mixed results [3,4]. The low recruitment rates of ethnic minority cancer survivors in cancer research remain a concern, and the low rates of participation also call into question the generalizability and applicability of extant findings and interventions for this population. There are substantial public health and societal benefits to be gained by identifying culturally sensitive and effective recruitment strategies for this population.

Asian American people are the fastest growing and Latinx American people are the largest ethnic minority population in the United States. Despite marked improvements in cancer prognosis and survival rates for the general population, Asian American and Latinx American people are more likely to experience poorer quality of life relative to non-Hispanic white people [5]. Recent research has also showed increases in the annual incidence rate of breast cancer among Asian American women [6]. There are many reasons for the low participation rate of ethnic minority cancer survivors in research. These reasons may include language barriers [7], low socioeconomic status, lack of health insurance and access to care [8], cultural barriers such as distrust of researchers [9], and stigmatized beliefs about cancer [10]. These barriers limit access to traditional recruitment methods that often rely on physicians to make referrals in health care settings.

With the widespread use of social media, health researchers have turned to Facebook as a potential source of recruitment [11]. As the most popular social media website, with 2.2 billion active users who have logged in at least once during the last 30 days [12], Facebook has qualities that may help overcome barriers and facilitate the recruitment of ethnic minority cancer survivors. For instance, the language barrier can be overcome by translating ads into different languages and targeting them to appear to users who are using Facebook in their native languages. With a potential to overcome barriers to recruitment in health care settings, there is evidence that Facebook is now widely used by adults aged ≥65 years and those with low socioeconomic status backgrounds [13]. Specifically, 62% of older adults who used the internet used Facebook, and 77% of people with an annual household income <US $30,000 used Facebook in 2016 [14,15]. These trends suggest that Facebook has a strong potential to reach ethnic minority cancer survivors who come from lower socioeconomic status backgrounds that may not otherwise have exposure to research opportunities. Lastly, in the privacy of their homes, potential participants who are less trusting of researchers can take the time they need to evaluate the benefits and costs of participating in research.

An increasing number of studies have demonstrated the feasibility of using Facebook to recruit participants for health and medical research [11]. However, to our knowledge, we are the first to evaluate the feasibility and effectiveness of paid advertisements on Facebook as a platform for identifying Chinese, Korean, and Latinx American cancer survivors. We assessed participants’ experience with participating in this Web-based survey study and their willingness to participate in future research studies.

## Methods

### Facebook Ads

**Figure 1:**
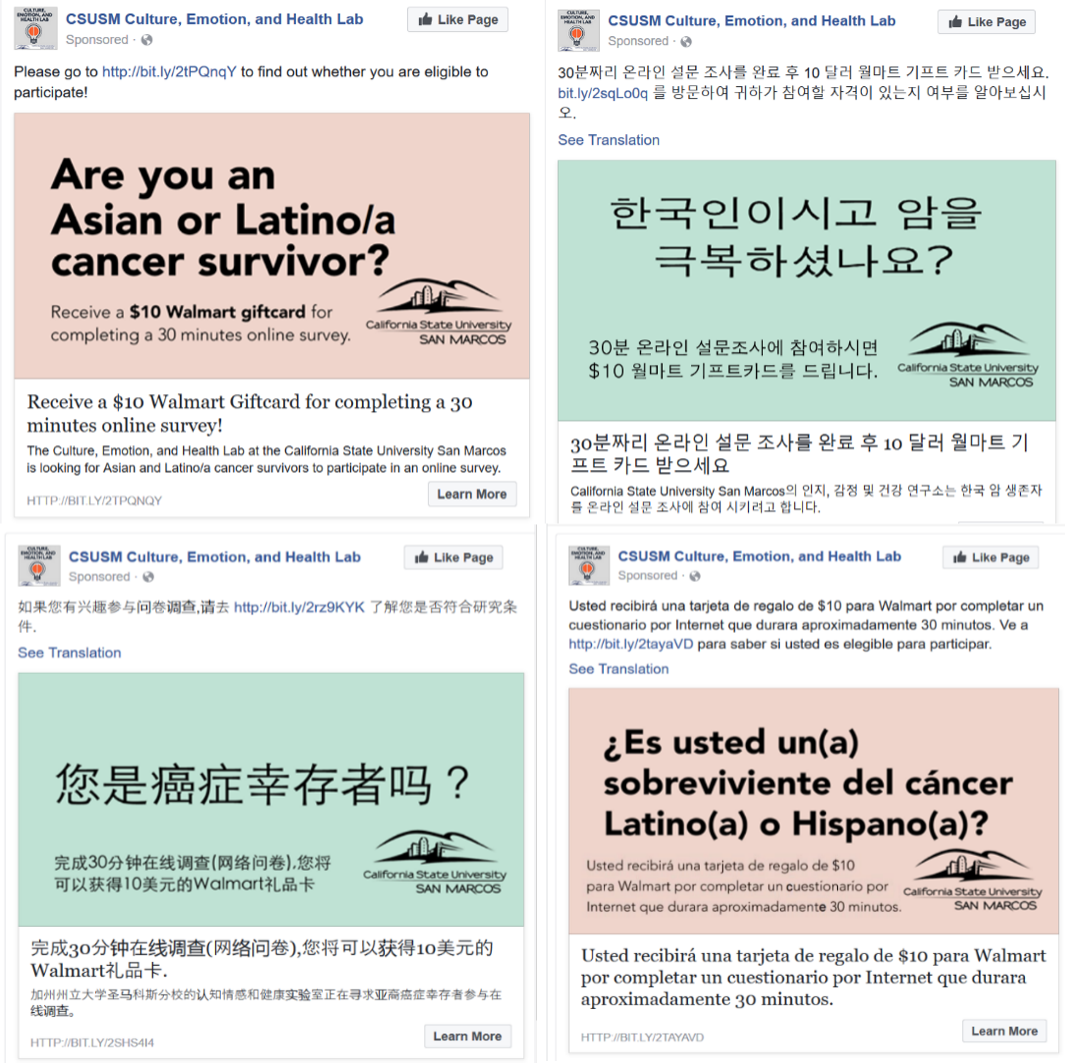
Example of Facebook ads. The simplified Chinese ad is not shown.

In total, we purchased 5 ads in English, simplified Chinese, traditional Chinese, Korean, and Spanish and showed them on Facebook from June 16 to August 3, 2017. The California State University San Marcos Institutional Review Board approved the ads shown on Facebook. Following the recommendations by Thornton et al [11], the ads were concise, showed the affiliation of the research study with a university, and offered incentives for participation (Figure 1). The link in each Facebook ad connected to the study website, which stated the eligibility criteria for participating in the 30-minute Web-based survey in the respective language of the clicked ads (eg, clicking the Korean ad would lead to the study information presented in Korean).

### Study Procedures

When participants clicked on the Facebook ad, they were directed to the study website that provided a general description of the study and the inclusion criteria. On this website, participants interested in the study submitted their email address by entering it into the “email” submission box. Then, participants received a more detailed description of the study as well as a link to the Web-based survey via email. We directed participants who accessed the survey link from their email inbox to the Web-based survey cover page that described the study, which explained the inclusion criteria, study procedure, and data confidentiality and stated that their consent was given by continuing with the survey. The first 5 questions screened potential participants for eligibility by asking whether they were Asian or Latinx, lived in the United States, aged ≥18 years, diagnosed with cancer, and within 5 years of their cancer treatment. Participants who answered “yes” to each of the 5 questions were directed to complete the survey, which assessed their demographic and cancer diagnosis information, well-being, experience with the study, and interest in participating in future research studies. We did not report the data on well-being in this study. Participants who completed the survey were sent a US $10 Walmart eGiftcard.

### Measures

#### Participation Experience

We assessed participants’ experience with the Web-based research study using 3 items rated on a 4-point Likert Scale ranging from 1 (“Strongly disagree”) to 4 (“Strongly agree”). We assessed the perceived difficulty with completing the research study using 2 items (ie, “I did not experience technical difficulty while participating in this research” and “Participating in this study was easy”). A composite perceived difficulty variable was created by averaging these 2 items. The third item assessed the extent to which they enjoyed having the opportunity to share their feelings and experiences with cancer through this research study (ie, “I enjoyed the opportunity to share my feelings and experience about cancer in this research study”). Higher scores indicate lower perceived difficulty with completing the research and greater enjoyment of having completed the survey.

#### Willingness and Access to Smartphone to Participate in Future Research

We assessed participants’ interest in future research studies using 4 items (eg, “I would like to participate in more research studies in the future”) rated on a 4-point Likert Scale ranging from 1 (“Strongly disagree”) to 4 (“Strongly agree”). The other 3 items assessed participants’ willingness to participate in a Web or phone interview with a research staff member, willingness to privately write about their cancer experiences, and willingness to privately voice record their cancer experiences. The 4 items were averaged, with higher scores indicating greater willingness to participate in future research (Cronbach alpha=.80 and.88 for Latinx and Asian cancer survivors, respectively). Lastly, we assessed whether the participant had a smartphone, as a means to participate in future research.

#### Advertising Campaign

The Facebook advertisement platform (ie, Facebook Ads Manager) provides researchers with the ability to target the age, location, interests, and behaviors of those who will be shown the ads. We targeted ads at Facebook users with interests in cancer-related keywords or organizations (eg, American Cancer Society, Oncology, Breast cancer care, Skin cancer or Melanoma awareness, National Cancer Institute, Susan G. Komen for the Cure, cancer awareness, and American Cancer Society Relay for Life) and behaviors related to cancer causes. In addition to the target interests and behaviors above, the English ad targeted participants who used Facebook in English, simplified or traditional Chinese, and Spanish and those who indicated interests in Hispanic or Asian culture. For the other translated ads, they were delivered to Facebook users who used that specific language and those who indicated interests or behaviors with an affinity to the respective culture (eg, the Spanish ad was delivered to participants who used Facebook in Spanish and those who indicated interests or behaviors related to Hispanic culture). All ads were delivered to adult Facebook users who lived in the United States.

## Results

### Advertisement Campaign

**Figure 2:**
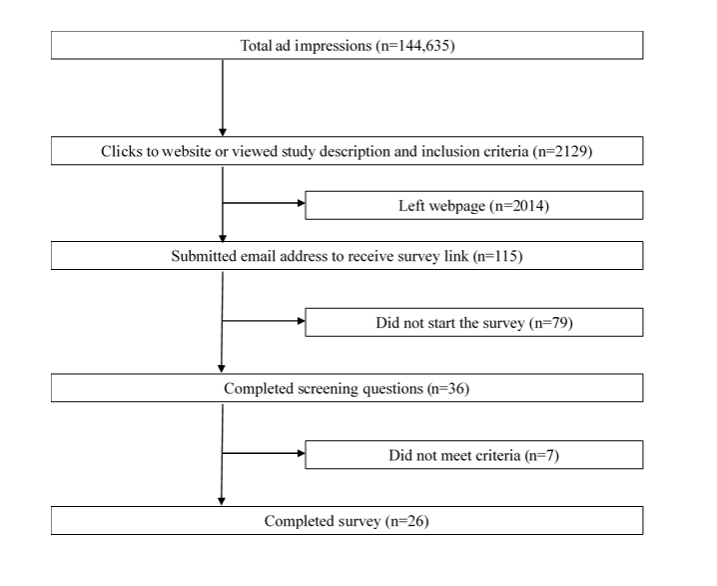
Flow diagram of impressions, ad clicks, email submissions, survey participation, and completion; 3 surveys were completed by a participant with the same internet protocol address and subsequently removed from analyses.

The ad campaign was set up with a maximum daily budget of US $25 and ran for 48 consecutive days for a total spending of US $1,200.46 (June 16 to August 3, 2017). In total, the English, Spanish, Korean, and traditional and simplified Chinese ads generated 45,768, 34,261, 30,335, 21,230, and 13,041 impressions (ie, total number of times the ads were shown to Facebook users) and reached 31,299, 25,217, 8187, 7183, and 4915 people (ie, number of unique Facebook users who saw the ads), respectively. These impressions generated 420, 984, 307, 291, and 127 link clicks for the English, Spanish, Korean, and traditional and simplified Chinese ads, respectively. The average cost per click across the 5 ads was US $0.56 (see Table 1). Of the 2129 total link clicks that directed the potential participant to the study website, 115 people entered their emails after reading the study information and eligibility criteria (Figure 2). Due to 10 incorrectly submitted emails, 10 survey links were not delivered. Of the105 people who were sent the Web-based survey link, 36 (34.3%) clicked on the survey link and completed the initial screening questions. Of these 36 people who begun the survey, 29 (81%) were eligible and completed the Web-based survey (10 Chinese, 4 Korean, and 15 Latinx cancer survivors); however, 3 (10%) surveys were completed by a Chinese participant with the same internet protocol address and, thus, were excluded from subsequent analyses. In total, data from 4 surveys in English (3 by Latinx and 1 by Chinese participants), 4 surveys in Korean, 5 surveys in traditional Chinese, 1 survey in simplified Chinese, and 12 surveys in Spanish were analyzed. The cost per participant was US $46.17 (ie, total spending per number of completed Web-based surveys).

### Participant Demographic and Cancer Diagnosis Information

We combined the Chinese and Korean participants into 1 East Asian participant group. Of the 11 East Asian cancer survivors (mean age 55.91 [SD 10.03] years; 8/11, 73%, females), 91% (10/11) were first-generation immigrants (ie, foreign-born), 55% (6/11) obtained a college degree, and 9% (1/11) reported an annual family income <US $13,000. The majority (9/11, 82%) spoke Chinese or Korean but 64% (7/11) also reported the ability to speak English. Most of the East Asian cancer survivors had diagnoses of breast cancer (5/11, 46%) or lymphoma (3/11, 27%), and 55% (6/11) were in remission. Of the 15 Latinx cancer survivors (mean age 49.73 [SD 9.71] years; 13/15, 87%, females), 80% (12/15) were first-generation immigrants, 53% (8/15) obtained a college degree, and 47% (7/15) reported an annual family income <US $13,000. The majority (13/15, 87%) spoke Spanish, but only 20% (3/15) also reported the ability to speak English. Most of the Latinx cancer survivors had diagnoses of breast cancer (7/15, 47%) or colorectal cancer (3/15, 20%). See Table 2 for complete demographic and cancer diagnosis information.

**Table 1:** Facebook advertisement campaign.

Ad language	Link clicks	Impressions (people)	Reach (people)	Cost per click (US $)	Amount spent (US $)
English	420	45,768	31,299	0.67	281.37
Spanish	984	34,261	25,217	0.31	307.97
Korean	307	30,335	8187	0.97	296.29
Simplified Chinese	127	13,041	4915	0.66	83.60
Traditional Chinese	291	21,230	7183	0.79	231.23
Total	2129	144,635	71,412^a^	0.56	1200.46

^a^This number reflects the total unique number of people who saw at least one ad.

We examined demographic differences between East Asian and Latinx cancer survivors. They did not significantly differ regarding age (*P*=.13), years lived in the United States (*P*=.10), education level (*P*=.92), generation status (*P*>.99), stage of cancer diagnosis (*P*=.35), time since diagnosis (*P*=.85), and treatment status (*P*=.82). However, Latinx cancer survivors were marginally more likely to only speak their heritage language (ie, limited English proficiency; *P*=.06) and marginally more likely to endorse an annual family income <US $13,000 compared with the East Asian cancer survivors (*P*=.09).

### Experience with the Web-Based Survey and Interest in Future Research

We examined participants’ experience with this study and their interest in participating in future research (Table 3). East Asian cancer survivors reported a lower ease of participation (eg, having technical difficulties accessing the survey) in this study than Latinx cancer survivors (mean 2.91 [SD 0.66] vs mean 3.47 [SD 0.52]; two-tailed *t*_24_=2.41; *P*=.02). Both Latinx and East Asian cancer survivors reported that they enjoyed the opportunity to share their cancer-related experiences in this Web-based survey (mean 3.40 [SD 0.63] and mean 3.27 [SD 0.47], respectively; two-tailed *t*_24_=0.37; *P*=.56). Both Latinx and East Asian cancer survivors indicated a willingness to participate in future cancer research studies through video or phone interviews with study researchers or sharing their cancer experiences through writing or voice recording (mean 3.00 [SD 0.54] and mean 3.00 [SD 0.63], respectively; two-tailed *t*_24_=0; *P*>.99). The majority of Latinx and East Asian cancer survivors indicated having access to a smartphone (13/15, 87%, and 10/11, 91%, respectively).

**Table 2:** Latinx and Asian cancer survivor demographic and cancer diagnosis information.

Variable		Latinx survivors (n=15)	East Asian survivors (n=11)	*P* value^a^
**Ethnicity, n (%)**				
	Mexican	8 (53)	—^b^	—
	Cuban	2 (13)	—	—
	Puerto Rican	4 (27)	—	—
	Honduran	1 (7)	—	—
	Mainland Chinese	—	3 (27)	—
	Taiwan Chinese	—	3 (27)	—
	Hong Kong Chinese	—	1 (9)	—
	Korean	—	4 (36)	—
Age (years), mean (SD)		49.73 (9.71)	55.91 (10.03)	.13
Time since diagnosis (months), mean (SD)		26.36 (12.64)	27.80 (25.10)	.85
**Gender, n (%)**				.62
	Male	2 (13)	3 (27)	—
	Female	13 (87)	8 (73)	—
**Education,** **n (%)**				.92
	Less than high school	3 (20)	1 (9)	—
	High school degree	2 (13)	1 (9)	—
	Some college	1 (7)	2 (18)	—
	College degree	8 (53)	6 (55)	—
	Professional or graduate degree	1 (7)	1 (9)	—
**Geographic location,** **n (%)**				.25
	South	7 (47)	2 (18)	—
	East	2 (13)	1 (9)	—
	Midwest	0 (0)	2 (18)	—
	Northeast	2 (13)	2 (18)	—
	West	4 (27)	4 (36)	—
**Generation status,** **n (%)**				
	First generation	12 (80)	10 (91)	>.99
	Second generation	2 (13)	1 (9)	—
	Fourth generation	1 (7)	0 (0)	—
**Language spoken,** **n (%)**				.06
	Heritage language only	12 (80)	4 (36)	—
	English only	2 (13)	2 (18)	—
	Both heritage and English	1 (7)	5 (45)	—
**Marital status,** **n (%)**				.60
	Single	2 (13)	1 (9)	—
	Married	10 (67)	7 (64)	—
	Divorced	1 (7)	2 (18)	—
	Separated	2 (13)	0 (0)	—
	Bereaved	0 (0)	1 (9)	—
**Annual household income,** **n (%)**				.09
	Less than US $13k	7 (47)	1 (9)	—
	US $13k to $30k	2 (13)	2 (18)	—
	US $30k to $60k	4 (27)	3 (27)	—
	US $60k to $100k	0 (0)	3 (27)	—
	Greater than US $100k	1 (7)	2 (18)	—
	Missing	1 (7)	0 (0)	—
**Stage of cancer diagnosis,** **n (%)**				.35
	I	4 (27)	1 (9)	—
	II	6 (40)	3 (27)	—
	III	2 (13)	2 (18)	—
	IV	0 (0)	2 (18)	—
	Missing	3 (20)	3 (27)	—
**Cancer type,** **n (%)**				.23
	Breast	7 (47)	5 (45)	—
	Blood	1 (7)	1 (9)	—
	Colorectal	3 (20)	0 (0)	—
	Oral	1 (7)	0 (0)	—
	Skin	1 (7)	0 (0)	—
	Thyroid	2 (13)	1 (9)	—
	Lymphoma	0 (0)	3 (27)	—
	Pancreatic	0 (0)	1 (9)	—
**Treatment status,** **n (%)**				.82
	Currently in treatment	5 (33)	5 (45)	—
	In remission	9 (60)	6 (55)	—
	Missing	1 (7)	0 (0)	—
**Cancer treatment,** **n (%)**				.91
	Chemotherapy only	2 (13)	1 (9)	—
	Radiation only	2 (13)	0 (0)	—
	Surgery only	2 (13)	0 (0)	—
	Medication only	1 (7)	2 (18)	—
	Chemotherapy, radiation, and surgery	5 (33)	5 (45)	—
	Chemotherapy and radiation	1 (7)	1 (9)	—
	Chemotherapy and surgery	1 (7)	1 (9)	—
	Radiation and surgery	1 (7)	1 (9)	—

^a^*P* values for categorical variables are based on chi-square or Fisher’s exact test, and *P* values for continuous variables are based on two-tailed *t* tests.

^b^Not applicable.

**Table 3:** Participant experience with this study and willingness to participate in future research.

Participant experience		Latinx (n=15)	East Asian (n=11)		*P* value^a^
**Perceived difficulty with study composite^b^,** **mean (SD)**		3.47 (0.52)	2.91 (0.66)		.02
	I did not experience technical difficulty while participating in this research.	3.40 (0.63)	2.82 (0.87)		.06
	Participating in this study was easy.	3.53 (0.52)	3 (0.78)		.05
**Participation enjoyment^b^, mean (SD)**					
	I enjoyed the opportunity to share my feelings and experience about cancer in this research study.	3.40 (0.63)	3.27 (0.47)		.56
**Interest in future research composite^b^, mean (SD)**		3.07 (0.43)	3.00 (0.50)		.72
	I would like to participate in more research studies in the future.	3.47 (0.52)	3.27 (0.47)		.34
	I would be willing to participate in a Web or phone interview with research staff.	2.93 (0.46)	3.09 (0.54)		.43
	I would be willing to write about my cancer experiences in a private journal.	3 (0.54)	3 (0.63)		>.99
	I would be willing to speak about my cancer experiences by recording into a voice recorder.	2.87 (0.64)	2.64 (0.67)		.38
**Access to a smartphone, n (%)**				.74	
	Yes	13 (87)	10 (91)		—^c^
	No	2 (13)	1 (9)		—

^a^*P* values for categorical variables are based on chi-square or Fisher’s exact test, and *P* values for continuous variables are based on two-tailed *t* tests.

^b^Likert ratings range from 1=“Strongly disagree” to 4=“Strongly agree”.

^c^Not applicable.

## Discussion

### Principal Findings

This study demonstrated the feasibility and effectiveness of recruiting Latinx and East Asian cancer survivors via targeted advertisements on Facebook. With a focus on identifying cancer survivors who may benefit from participating in psychosocial intervention research (ie, within 5 years of cancer diagnosis), we examined their experience with our Web-based survey and their interest in participating in future research.

We found that the Facebook campaign was successful in recruiting a sample of Latinx and East Asian cancer survivors with different cancer diagnoses from various geographic regions of the United States. Latinx and East Asian cancer survivors recruited from Facebook enjoyed sharing their cancer experiences and reported their interest in participating in future psychosocial intervention studies ranging from Web-based focus groups to private emotional disclosure through writing or voice recording (eg, expressive writing interventions [16]). Furthermore, we found that all but 3 (23/26, 88%) participants had access to smartphones and, thus, could participate in Web-based or mobile-based psychosocial interventions that have become increasingly popular in recent years [17].

Most of the Latinx and East Asian cancer survivors were immigrants, female, and had breast cancer diagnoses. However, Latinx cancer survivors were more likely to speak only their heritage language (eg, Spanish) and reported a lower annual household income than the East Asian cancer survivors. These findings suggest that Facebook may be an especially effective source of recruitment for immigrant Latinx cancer survivors who are from lower socioeconomic backgrounds. Because this population represents a significantly understudied and underserved population with significant cancer health disparities [5], our findings build upon a growing number of studies that have demonstrated the success of using Facebook to recruit harder-to-reach medical populations [18].

A significant portion of potential participants did not participate in the Web-based survey after receiving the survey link in their emails. Although speculative, these participants may have submitted their emails on the spur of the moment after clicking the Facebook advertisement without carefully reading the study description and inclusion criteria on the website, and only later learned from the received email that they were ineligible. Alternatively, it is also possible that they had difficulty trusting the researchers and the email they received [9]. Although we included the affiliated university logo where possible (eg, on the Facebook ad and study website), the research team’s email used to send the survey link was not from a university-affiliated email address. Perhaps using a university-affiliated email address would have increased the participants’ trust and willingness to complete the survey. Nonetheless, we successfully recruited 11 East Asian and 15 Latinx cancer survivors in 48 consecutive days of recruitment. Notably, we only recruited through the paid Facebook advertisement platform and did not utilize other opportunities that were freely available on Facebook. For example, we did not manually post our ads on cancer-related Facebook pages and groups [19]. Researchers considering Facebook to recruit ethnic minority cancer survivors can potentially maximize their success by utilizing both free and paid advertisement strategies.

### Limitations

While we found support for the feasibility and effectiveness of recruiting East Asian and Latinx cancer survivors from Facebook, our study has several limitations. First, we did not include a direct comparison with traditional methods of recruitment (eg, physician referrals or flyering), so we could not determine whether Facebook recruitment is a more efficient or cost-effective method than traditional methods. However, the costs of hiring research staff with language abilities to distribute flyers and screen potential participants are likely more expensive and time-intensive than the minimal time and personnel needed for recruitment through Facebook. Although we cannot speculate on the demographic similarities or differences between the East Asian and Latinx cancer survivors recruited through Facebook or in person, a growing number of studies have found minimal differences between Web-based and in-person recruitments [11]. However, the feasibility and effectiveness of recruiting through other social media platforms (eg, Craigslist) requires future testing. Moreover, given that the study was conducted through the Web, we could not verify the veracity of the information provided by the participants. It is also unclear whether participants who clicked on the ads and viewed the study page were eligible but not interested in participating or whether they were ineligible owing to having completed cancer treatment over 5 years ago. Lastly, given the focus on Chinese and Korean cancer survivors in this study, the generalizability of our findings to other Asian subgroups (eg, Filipino and Hmong) from Facebook remains to be tested.

### Conclusions

The recruitment of ethnic minority cancer survivors into cancer research has been a longstanding issue. With the potential to address this problem, we found that Facebook was a feasible and effective platform for recruiting Latinx and East Asian cancer survivors who may be interested in future psychosocial intervention research. Facebook was successful in reaching participants with different cancer diagnoses who come from various geographic regions. Ethnic minority cancer survivors are regarded as a challenging population to recruit, partially owing to limited English proficiency and low socioeconomic status. The Latinx and East Asian cancer survivors who participated in this study reported favorable experiences with the Web-based survey and reported interest in participating in future research studies. Future research studies should compare demographic and psychosocial differences between participants recruited from traditional and Web-based sources.
